# Tumour in the dark: a challenging case of osteomalacia

**DOI:** 10.1093/omcr/omae159

**Published:** 2024-12-28

**Authors:** Robert Ambrogetti, Omer Taha, Mohamed Saeed, Prashanth Patel, Faizanur Rahman

**Affiliations:** Department of Medicine, University Hospitals of Leicester NHS Trust, Infirmary Square, Leicester LE15WW, United Kingdom; Department of Medicine, University Hospitals of Leicester NHS Trust, Infirmary Square, Leicester LE15WW, United Kingdom; Department of Chemical Pathology & Metabolic Diseases, University Hospitals of Leicester NHS Trust, Groby Road, Leicester LE39QP, United Kingdom; Department of Chemical Pathology & Metabolic Diseases, University Hospitals of Leicester NHS Trust, Groby Road, Leicester LE39QP, United Kingdom; Department of Chemical Pathology & Metabolic Diseases, University Hospitals of Leicester NHS Trust, Groby Road, Leicester LE39QP, United Kingdom

**Keywords:** osteomalacia, tumour-induced osteomalacia, paraneoplastic, FGF23

## Abstract

Tumour-induced osteomalacia (TIO), also known as oncogenic osteomalacia, is a rare paraneoplastic syndrome mediated by the overproduction of phosphaturic hormone fibroblast growth factor 23. TIO is most commonly caused by mesenchymal tumours (PMTs), which are typically small, slow-growing and often undetectable on physical examination and conventional imaging techniques. Patients with TIO typically undergo a protracted period of diagnostic workup and medical treatment due to presentation with nonspecific symptoms and difficulty in localising the culprit tumour. During this period, ongoing surveillance is imperative as medical treatment can limit symptom progression, and tumour identification can provide definitive treatment. We report a case of TIO secondary to a PMT, which, despite biochemical diagnosis, medical treatment and serial imaging, took approximately ten years for tumour localisation.

## Introduction

Tumour-induced osteomalacia (TIO) is a rare paraneoplastic syndrome mediated by tumour overproduction of phosphaturic hormone fibroblast growth factor 23 (FGF23) [1]. Patients with TIO typically present with nonspecific symptoms of progressively worsening and unexplained chronic pain, proximal muscle weakness, recurrent poorly healing fractures, chronic fatigue, and associated accumulation of disability [1]. TIO is most commonly caused by mesenchymal tumours (PMTs) [2]. PMTs are characterised by paraneoplastic FGF23 overproduction, leading to renal phosphate wasting, chronic hypophosphatemia and, ultimately, osteomalacia [1]. Due to difficulties in localising a culprit tumour, many patients have a protracted period of medical management [1]. PMTs have been reported to occur at any age, however predominantly manifest in later life [2]. We report a case of TIO secondary to an underlying PMT. Even though a diagnosis was made based on clinical and laboratory investigations, it took approximately ten years for the tumour to be found.

## Case report

A man in his 50s of Indian background was referred to the metabolic bone clinic with a five-year history of painful limbs, refractory hypophosphataemia, and elevated alkaline phosphatase despite the correction of severe vitamin D deficiency initially identified. During this time, he had been followed up by medical services however no firm diagnosis had been established.

He had a past medical history of spinal decompression two years earlier and two previous episodes of nephrolithiasis. He had no family history of metabolic or bone disease and no prior steroid use. He reported increasing weakness and paraesthesia in his upper and lower limbs, progressing to mostly requiring a wheelchair. No bony lesions or lymphadenopathy were identified on examination.

Initial biochemistry showed low adjusted calcium, inorganic phosphate, 25-OH vitamin D, and elevated alkaline phosphatase (ALP) with isoenzymes predominantly from bone (Table 1). 1-25diOH Vitamin D was in the low normal range. His PTH, PSA, thyroid function, full blood count, electrolytes, renal function, coeliac and myeloma screen were normal. His ferritin, vitamin B12 and folate levels were optimised with supplementation. The urinary amino acid profile was normal. However, fractional excretion of urinary phosphate was elevated at 27%, and the ratio of tubular maximum reabsorption of phosphate to GFR of 0.42 (0.8–1.35) was noted.

**Table 1:** 

**Investigations**				
	**Baseline**	**Post medical treatment**	**Post PMT resection**	**Normal Values**
WBC	7.8			4.0–11.0 × 109/L
Haemoglobin	141			130–180 g/L
eGFR	>90			mL/min/1.73 m2
Creatinine	67			60–120 umol/L
Na	140			133–146 mmol/L
K	5.5			3.5–5.3 mmol/L
TSH	1.1			0.30–5.00 miu/L
PTH	6.4			1.6–7.5 pmol/L
B12	360			220–700 ng/L
Folate	5.2			ug/L
Urea	8.9			2.5–7.8 mmol/L
Ca(a)	2.06	2.48	2.3	2.2–2.6 mmol/L
Pi	0.48	1.08	1.09	0.8.1.5 mmol/L
ALP	628	106	54	30–130 iu/L
Bone ALP	478	–	–	14–44 iu/L
25—OH Vitamin D	<15 nmol/L	80	84	Nmol/L
1,25, diOH Vitamin D	39	114	55	20–120 pmol/L
FGF23	17 080	2500	78	0.0–100.0 RU/ml
**Other**				
Coeliac screen	negative			
PSA	0.9			
Blood film	normal			
Myeloma screen	negative			
Urine Amino acid profile	no abnormality detected			
Beta crosslaps	1.31			<0.64 ug/L

Key: Ca(a) = Adjusted calcium.

**Figure 1:**
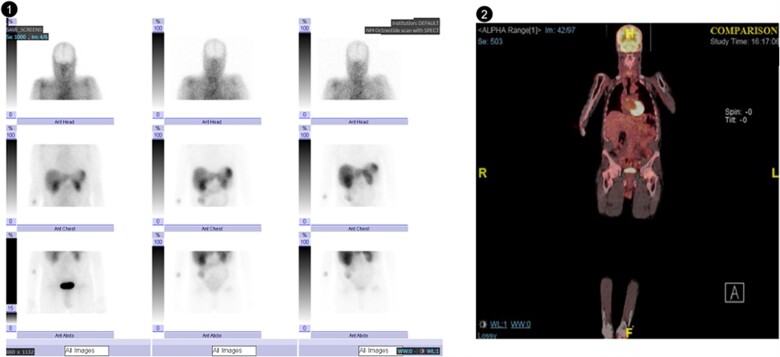
(1) Octreotide scans with single-photon emission computed tomography showing physiological uptake in the liver, spleen, kidneys and large bowel. The focal area of uptake related to the right area corresponds to the isotope injection site. No focal abnormal area of uptake was demonstrated. (2) Fluorodeoxyglucose-18 (FDG) positron emission tomography (PET) scan showing no abnormal activity.

Computer tomography of the thorax, abdomen and pelvis did not identify any malignancy but did show multiple fractures of the ribs, pelvic bones, neck of femur, and left scapular, with multiple sclerotic lesions in keeping with osteomalacia. Bone mineral density scan revealed a T score of −4.7 at L1-L4, a T score of −3.7 in the left femoral neck and a T score of −5.3 in the right femoral neck. FGF23 level was elevated at 17080 RU/ml (0.0 to 100.0 RU/ml). As a result, TIO was suspected. Initial whole-body PET-FDG (including skull and extremities) and octreotide scans did not localise any culprit malignant process (Fig. 1).

The patient had two further negative FDG PET scans over the next four years. His FGF23 (2500–17 080 RU/ml) remained elevated in keeping with TIO. Alfacalcidol 1ug once daily, vitamin D 1000 units once daily, calcium (AdcalD3 1 tablet twice daily) and phosphate (Sandoz Phos 1 tablet thrice daily) were used to manage hypocalcaemia and hypophosphatemia with some response (Table 1).

**Figure 2:**
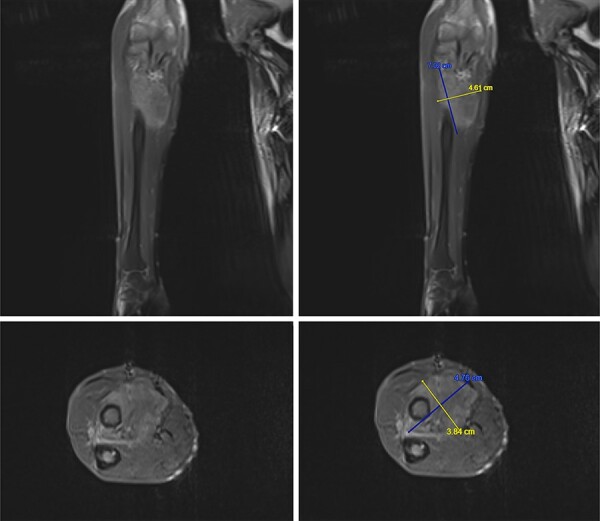
MRI T1 Turbo-spin-echo (TSE) of the right forearm showing the intermuscular enhancing lesion at the volar aspect of the proximal radius distal to the radial tuberosity interposed deep to the flexor and extensor muscles. The lesion measures approximately 7 cm × 4.6 cm × 3.84 cm.

**Figure 3:**
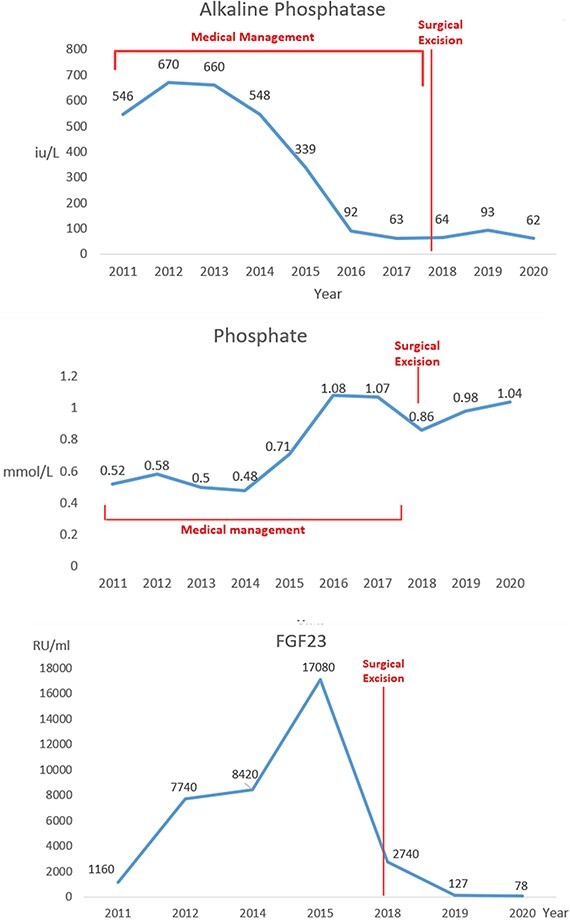
Trends of phosphate, FGF23 and alkaline phosphate throughout the clinical course.

Approximately two years after the last negative FDG PET scan, six years after the biochemical diagnosis of TIO and ten years from symptom onset, the patient noted a right forearm lump. On MRI, the lesion was identified as a 7 × 4 × 3 cm solid intramuscular lesion (Fig. 2). Histology confirmed spindle cell appearance with immunohistochemistry positive for CD34, BCL2, CD99 and SMA and negative for STAT6, SOX10, Desmin, S100 protein, AE1/3 EBER and MUC4. Since excision of the tumour, the patient has had clinical and biochemical resolution. FGF23, ALP, phosphate and calcium levels normalised postoperatively (Fig. 3, Table 1). Supplementation and treatment with alphacalcidol were subsequently tapered off successfully. The patient has since regained his independence and remained stable on only a bisphosphonate for bone protection.

## Discussion

With non-specific symptoms, there is often a delay in the diagnosis of TIO, as demonstrated in our case [2]. Initial misdiagnosis rate has been reported to be as high as 95% [3].

A systematic review of 895 cases found that over 80% are diagnosed radiographically two years after symptom onset [4]. Our case highlights the importance of a biochemical diagnosis in cases where imaging does not detect any tumour for definitive surgical treatment. A biochemical diagnosis allows early medical intervention, which, in our case, mitigated functional decline. Burosumab, a monoclonal antibody which inhibits FGF23, has been shown to increase serum phosphate, improve osteomalacia and enhance the healing of fractures in the medical management of TIO [5]. Similarly, Cinacalcet has previously been used for medical management of TIO cases where no causative tumour for resection was located [6]. Cinacalcet is hypothesized to mitigate the effects of paraneoplastic FGF23 in TIO by activating parathyroid gland calcium-sensing receptors, inducing hypoparathyroidism, resulting in increased renal retention of phosphate [6].

Causative tumours are often small, slow growing and can be located anywhere on the body [4]. TIO-associated tumours are most commonly found in the lower limbs (46%) and head and neck (28%) [4]. Only 7% of TIO cases are associated with an upper limb tumour, as seen in our case, highlighting the importance of full-body imaging [4]. Functional imaging with somatostatin receptor-based PET/CT imaging has been shown to have the highest sensitivity (sensitivity 86%, 95% CI: 79%–91%) for identifying TIO-causing cancers [1, 2, 7, 8].

PMTs arising from soft tissue, as seen in our case, are associated with a worse prognosis as they can arise anywhere in the body, making them more difficult to detect [7]. Complete surgical resection is the treatment of choice and often definitive. Hence, the ability to completely resect the tumour is arguably the most critical factor related to long-term prognosis [1, 9]. As seen in our patient, surgical resection has resulted in significant biochemical and clinical improvement.

## Conclusion

In conclusion, TIO is an uncommon and frequently misdiagnosed condition which has potentially curative treatment. Our case demonstrates the culprit tumour may become detectable many years after symptom onset. This highlights the role of biochemical diagnosis for early treatment and the importance of extended follow-up to ensure patients do not miss potentially curative surgical intervention.
